# Diagnostic performance of image navigated coronary CMR angiography in patients with coronary artery disease

**DOI:** 10.1186/s12968-017-0381-3

**Published:** 2017-09-11

**Authors:** Markus Henningsson, Joy Shome, Konstantinos Bratis, Miguel Silva Vieira, Eike Nagel, Rene M. Botnar

**Affiliations:** 1 Division of Biomedical Engineering and Imaging Sciences, King’s College London, London, UK; 2 Institute for Experimental and Translational Cardiovascular Imaging, Goethe University, Frankfurt/Main, Germany; 3 DZHK (German Centre for Cardiovascular Research, Standort RheinMain), Berlin, Germany; 4 Escuela de Ingeniería, Pontificia Universidad Católica de Chile, Santiago, Chile

**Keywords:** Coronary MR angiography, Image navigators, Respiratory motion correction, Coronary artery disease

## Abstract

**Background:**

The use of coronary MR angiography (CMRA) in patients with coronary artery disease (CAD) remains limited due to the long scan times, unpredictable and often non-diagnostic image quality secondary to respiratory motion artifacts. The purpose of this study was to evaluate CMRA with image-based respiratory navigation (iNAV CMRA) and compare it to gold standard invasive x-ray coronary angiography in patients with CAD.

**Methods:**

Consecutive patients referred for CMR assessment were included to undergo iNAV CMRA on a 1.5 T scanner. Coronary vessel sharpness and a visual score were assigned to the coronary arteries. A diagnostic reading was performed on the iNAV CMRA data, where a lumen narrowing >50% was considered diseased. This was compared to invasive x-ray findings.

**Results:**

Image-navigated CMRA was performed in 31 patients (77% male, 56 ± 14 years). The iNAV CMRA scan time was 7 min:21 s ± 0 min:28 s. Out of a possible 279 coronary segments, 26 segments were excluded from analysis due to stents or diameter less than 1.5 mm, resulting in a total of 253 coronary segments. Diagnostic image quality was obtained for 98% of proximal coronary segments, 94% of middle segments, and 91% of distal coronary segments. The sensitivity and specificity was 86% and 83% per patient, 80% and 92% per vessel and 73% and 95% per segment.

**Conclusion:**

In this study, iNAV CMRA offered a very good diagnostic performance when compared against invasive x-ray angiography. Due to the short and predictable scan time it can add clinical value as a part of a comprehensive CAD assessment protocol.

## Background

Whole-heart coronary magnetic resonance angiography (CMRA) allows for non-invasive and ionizing radiation free detection of lumen narrowing coronary artery disease (CAD) [1]. Nevertheless, CMRA in patients with CAD remains limited due to the long scan times as well as unpredictable and often non-diagnostic CMRA image quality. The most common image degradation in CMRA is caused by image blurring and ghosting from respiratory motion [2]. This is due to the necessity of acquiring high-resolution whole-heart CMRA during free-breathing. Conventional motion compensation for CMRA involves interleaving a one-dimensional diaphragmatic ‘navigator’ acquisition to track the lung-liver interface in foot-head direction. This allows prospective correction (typically assuming a fixed linear relationship between motion of the diaphragm and that of the heart) and gating, which narrows down the range of acceptable motion to end-expiration at the expense of prolonging scan time [3].

In the last decade, a number of navigator techniques have been described which allow direct measurement and correction of respiratory induced motion of the heart. These include self-navigation, which extract the motion information from the CMRA data itself [4, 5], and image-based navigation where real-time images are used to estimate bulk respiratory motion of the heart [6–9]. In addition to directly tracking respiratory motion of the heart, self-navigation simplifies CMRA ease-of-use compared to other navigator approaches, as no dedicated navigator scan planning is necessary. Self-navigation and image-based navigation can be combined with affine [10, 11] and non-linear [12, 13] correction which aims to correct for all respiratory motion, leading to CMRA data across the whole respiratory cycle and shorter scan time compared to a gated scan. However, these advanced correction strategies require computationally expensive offline post-processing.

More recently, respiratory motion compensation using image-based navigation (iNAV) has been proposed for CMRA, and allows for accurate, direct tracking of the respiratory motion of the heart and can be implemented with inline correction [14]. In conjunction with efficient respiratory gating such as constant respiratory efficiency using single end-expiratory threshold (CRUISE), CMRA can be acquired with high image quality, with inline processing and in a clinically acceptable scan time [15].

The purpose of this study was to evaluate iNAV-CRUISE motion compensation for CMRA and compare it to gold standard invasive x-ray coronary angiography in patients with CAD.

## Methods

### Patient selection

Between February 2014 and October 2014, based on the availability of the research team, consecutive patients referred for CMR were considered for inclusion in this prospective study. The study was approved by the institutional ethics committee and all participants provided written informed consent. Patients were excluded from the study if they had pace makers, defibrillators or other general contraindications to CMR such as claustrophobia.

### CMR protocol

All experiments were performed on a 1.5 T clinical CMR scanner (Achieva, Philips Healthcare, Best, The Netherlands) using a 32-channel cardiac coil. The patients underwent a protocol consisting of multi-slice cine stack, first-pass perfusion, multi-slice late gadolinium enhancement stack and iNAV CMRA. For the late gadolinium enhancement, contrast medium was used (Gadobutrol, Gadovist®, Bayer AG, Leverkusen, Germany, dose: 0.2 mmol/kg). The CMRA scan was performed after contrast administration using bolus injection for the first-pass perfusion and before the late gadolinium enhancement scans. No specific patient preparation was performed, such as administration of β-blockers or nitroglycerine, for the CMRA scan.

### Image navigated CMRA

Image navigator correction and gating was implemented for CMRA respiratory motion compensation. The iNAV was acquired using 10 startup echoes of the balanced steady state free precesion (bSSFP) sequence, as previously described [16]. A region-of-interest encompassing the whole heart was tracked in foot-head (FH) and left-right (LR) direction, and selected using the local shim geometry. The iNAV reference was defined as the first acquired navigator to which all subsequent iNAVs were registered using normalised cross-correlation. The 2D translational correction was applied to the CMRA k-space raw data by modulating its phase. Respiratory gating was implemented using CRUISE. In brief, this approach acquires twice as much data as needed to fill CMRA k-space (resulting in exactly 50% gating efficiency) and only the half acquired at the most end-expiratory was used to reconstruct the gated image [15]. Both iNAV correction and gating was performed in real-time on the scanner, and no post-processing was required.

The CMRA protocol consisted of a bSSFP sequence with the following imaging parameters: FOV = 330 × 330 × 110 mm^3^, Δx = 1.3 × 1.3 × 1.3 mm^3^, repetition time/echo time = 3.9/1.95 ms, flip angle = 70°, coronal orientation, and parallel imaging acceleration factor = 2.5 (in-plane phase encoding direction). Electrocardiogram triggering was used to minimize cardiac motion, with subject-specific trigger delays and acquisition windows. To improve CMRA contrast, T2 prep (echo time = 35 ms) and fat suppression pre-pulses were used. The nominal scan time, including respiratory gating with 50% efficiency, was 7 min and 16 s, assuming a heart rate of 60 beats per minute and an acquisition window of 120 ms.

### Image analysis

All CMRA images were reformatted using dedicated software to visualize the right coronary artery (RCA), left main and left anterior descending coronary artery (LAD), and left circumflex coronary artery (LCX). To objectively and subjectively assess CMRA image quality, vessel sharpness measurements and visual score were performed on all datasets. Vessel sharpness was calculated on the first 4 cm of all coronary arteries, as a percentage where 0% equals no edge and 100% a step edge, using dedicated software [17]. For the patient data, vessel sharpness was assessed by an expert (Reviewer 1, with 8 years of experience in CMRA). Vessel sharpness was repeated on 10 random datasets 3 months later to assess intra-observer variability, compounding the vessel sharpness of the RCA, LAD and LCX. A second expert (Reviewer 2, with 4 years of experience in CMRA) performed vessel sharpness measurements on 10 random patient datasets to evaluate inter-observer variability, again, compounding sharpness values from the three coronary arteries.

A visual score was used, based on a previous CMRA patient study [1], to qualitatively assess coronary artery image quality based on the following scale: 0 – coronary artery not visible, 1 – visible but with marked blurring, 2 – visible with moderate blurring, 3 – visible with mild blurring, and 4 – visible with sharp edges. Visual score of 2 or higher was considered diagnostic quality. A segmental analysis was performed using a 9 coronary segment model, previously used in CMRA studies [18]. With this model the following segments were analysed: the left main (LM) artery, proximal, mid and distal segments of the LAD, proximal and mid segments of the LCX, and proximal mid and distal segments of the RCA. Coronary segments were excluded from analysis if they had previous stents or if the diameter was less than 1.5 mm. The visual scoring was performed independently by two experts, blinded to the patient’s information.

To assess diagnostic performance significant coronary stenosis was visually defined as luminal narrowing of more than 50% in each of the 9 segments using an intention-to-treat approach. The findings from the diagnostic reading of the coronary segments were compared to gold standard coronary x-ray angiographies, which were performed within 6 months of the CMRA scan. The diagnostic reading was performed by two expert readers, blinded to the x-ray angiography results. Disagreements between the readers were settled with a consensus reading. The likelihood of stenosis was graded according to the following scale: 1 – absent, 2 – probably absent, 3 – possibly present, 4 – probably present and 5 – definitely present [19].

### Statistical analysis

All statistical analyses were performed using MATLAB (The Mathworks Inc., Natick, MA USA) statistics toolbox. For the continuous variables vessel sharpness and scan time, a two-tailed t-test was performed to evaluate statistical significance. Continuous variables are presented as mean ± standard deviation. For the categorical variable (visual score) a Wilcoxon signed rank test was performed to evaluate statistical significance. Categorical variables are presented as median, 75th percentile, 25th percentile. A *P* value smaller than 0.05 was considered statistically significant. To evaluate intra-and inter-observer variability intra-class correlation coefficient was calculated for the different measurements. Additionally, the mean difference and standard deviation for the intra and inter-observer measurements were calculated. Inter-observer agreement of the visual scores was performed using Cohen’s kappa coefficient where a coefficient less than 0.4 was considered poor, between 0.4 and 0.75 good, and higher than 0.75 excellent agreement.

The visual scores for coronary segments were divided into proximal (proximal RCA, LM, proximal LAD, and proximal LCX), middle (middle RCA, LAD and LCX) and distal (distal RCA and LAD) segments to evaluate image quality between segments. This analysis included a Kruskal-Wallis one-way analysis of variance to determine any difference between the three groups, using a *P* < 0.05 to signify statistical difference. If a statistically significant difference was found, post hoc multiple Mann Whitney U tests were performed with a *P* < 0.017 considered statistically significant. The smaller significance threshold is due to the Bonferroni correction for multiple comparisons (0.05/3 = 0.017).

To assess whether patient variables such as age, heart rate, and body mass index (BMI) were correlated with vessel sharpness, bivariate analysis was performed. A linear regression model was calculated and Pearson’s correlation coefficient calculated to investigate if these variables could predict coronary vessel sharpness. The coronary vessel sharpness score was averaged across the RCA, LAD and LCX for each patient to obtain a single vessel sharpness score.

## Results

**Fig. 1:**
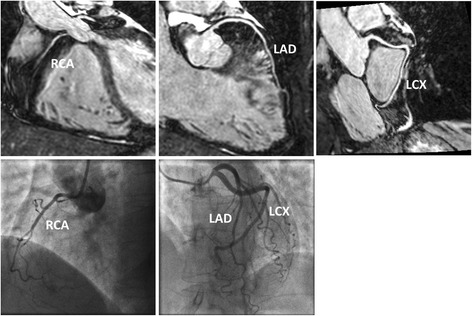
Reformatted CMRA datasets (top row) from a patient without coronary artery disease but non dominant right coronary artery (RCA). Coronary x-ray angiography in the same patient (bottom row). LAD = left anterior descending artery; LCX = left circumflex artery

In total, 31 patients were recruited for the study and their characteristics are summarized in Table 1. Of the 31 CMRA datasets, coronary stents precluded analysis in 8 coronary arteries. In total, out of a possible 279 coronary segments, 26 segments (8 proximal, 9 middle and 9 distal segments) were excluded from analysis due to stents or diameter less than 1.5 mm, resulting in a total of 253 coronary segments. The effective duration for the 31 patient scans was 7:21 ± 0:28 (min:sec). The CMRA acquisition was performed in systole in 19% of 6 patients (6 of 31) (heart rate = 78 ± 12 beats/min; acquisition window = 85 ± 22 ms; imaging time = 8:30 ± 0:33 min:sec) and diastole in 81% of patients (25 of 31) (heart rate = 63 ± 15 beats/min; acquisition window = 118 ± 38 ms; imaging time = 7:13 ± 0:18 min:sec). An example CMRA dataset from a patient without CAD but non dominant RCA which precluded analysis of mid and distal segments of the RCA is shown in Fig. 1.

**Table 1:** Patient characteristics

Total no of patients	31
Age (y)	56.4 ± 14.7
Men	24 (77.4%)
Heart rate (bpm)	66.4 ± 10.9
BMI (kg/m^2^)	27.3 ± 4.0
Hypertension	17 (55.8%)
Hyperlipidaemia	13 (41.9%)
Smoker	10 (32.2%)

### CMRA image quality

**Fig. 2:**
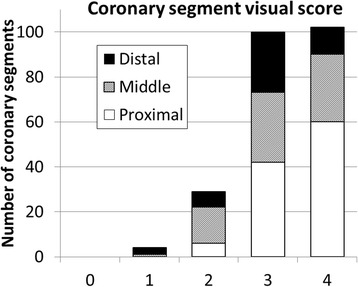
Distribution of visual scores of coronary segments, partitioned into proximal, middle and distal segments. A score of 0 is considered a non-visible coronary segment and 5 a visible segment with sharp edges. Visual scores of 2 or higher are considered to be of diagnostic image quality

The distribution of the visual scores of the 253 coronary segments, divided into proximal, middle and distal segments is shown in Fig. 2. The Kruskal-Wallis test, comparing distributions of visual scores for proximal, middle and distal segments, revealed a statistically significant difference (*P* < 0.01). Post-hoc Mann Whitney U test showed a statistically significant difference between visual scores for proximal and mid segments (proximal: 4,4,3 vs mid: 3,4,3, *P* < 0.01) and proximal and distal segments (proximal: 4,4,3 vs distal: 3,3,3 *P* < 0.001). Diagnostic image quality, defined as having a visual score of 2 or more, was obtained in 98% of all proximal coronary segments (113/115), 94% of middle segments (79/84), and 91% of distal coronary segments (49/54). In two patients, with significant arrhythmia, non-diagnostic image quality was found in 9 coronary segments, which corresponded to 75% of the total number of non-diagnostic segments. There was a good agreement between observers for the visual scoring, with a kappa coefficient of 0.71.

**Fig. 3:**
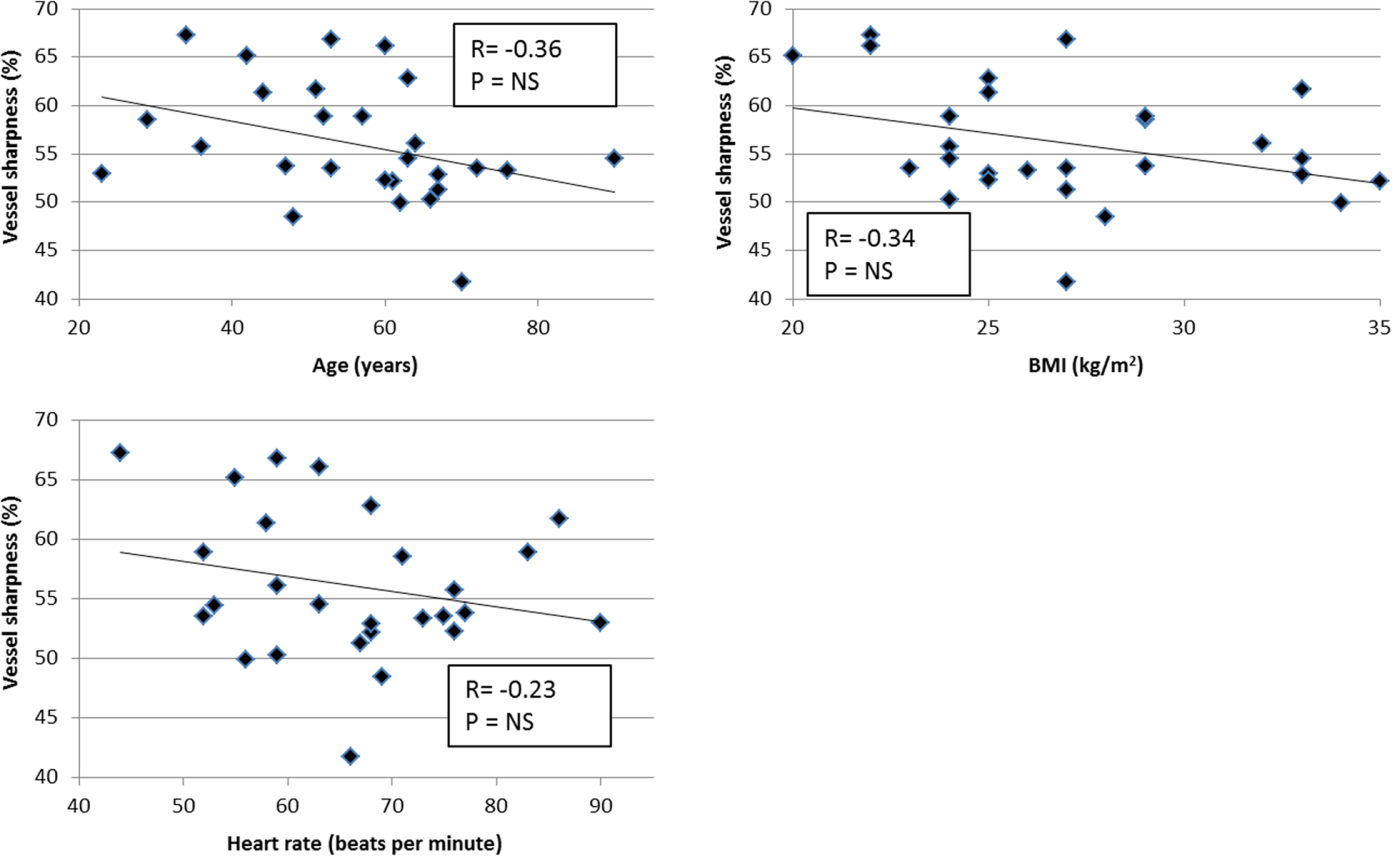
Scatter plots of coronary vessel sharpness versus age, body mass index (BMI), and heart rate. No statistically significant results were found for any of the correlations. NS = not significant

Vessel sharpness for the RCA was 53.9% ± 9.5%, LAD 56.2% ± 7.2% and LCX 51.9% ± 6.9%. Both intra- and inter-observer variability showed good agreement. The intra-observer mean difference was found to be −0.05% with a 95% confidence interval of 1.8% to −1.9%. The inter-observer mean difference was 0.23% and 95% confidence interval of 3.2% to −2.8%. The correlation analysis between patient characteristics and coronary vessel sharpness is shown in Fig. 3. None of the variables including age, BMI or heart rate predicted coronary sharpness on the multiple regression analysis (R^2^ = 0.10, p = N.S.).

### Diagnostic performance

**Fig. 4:**
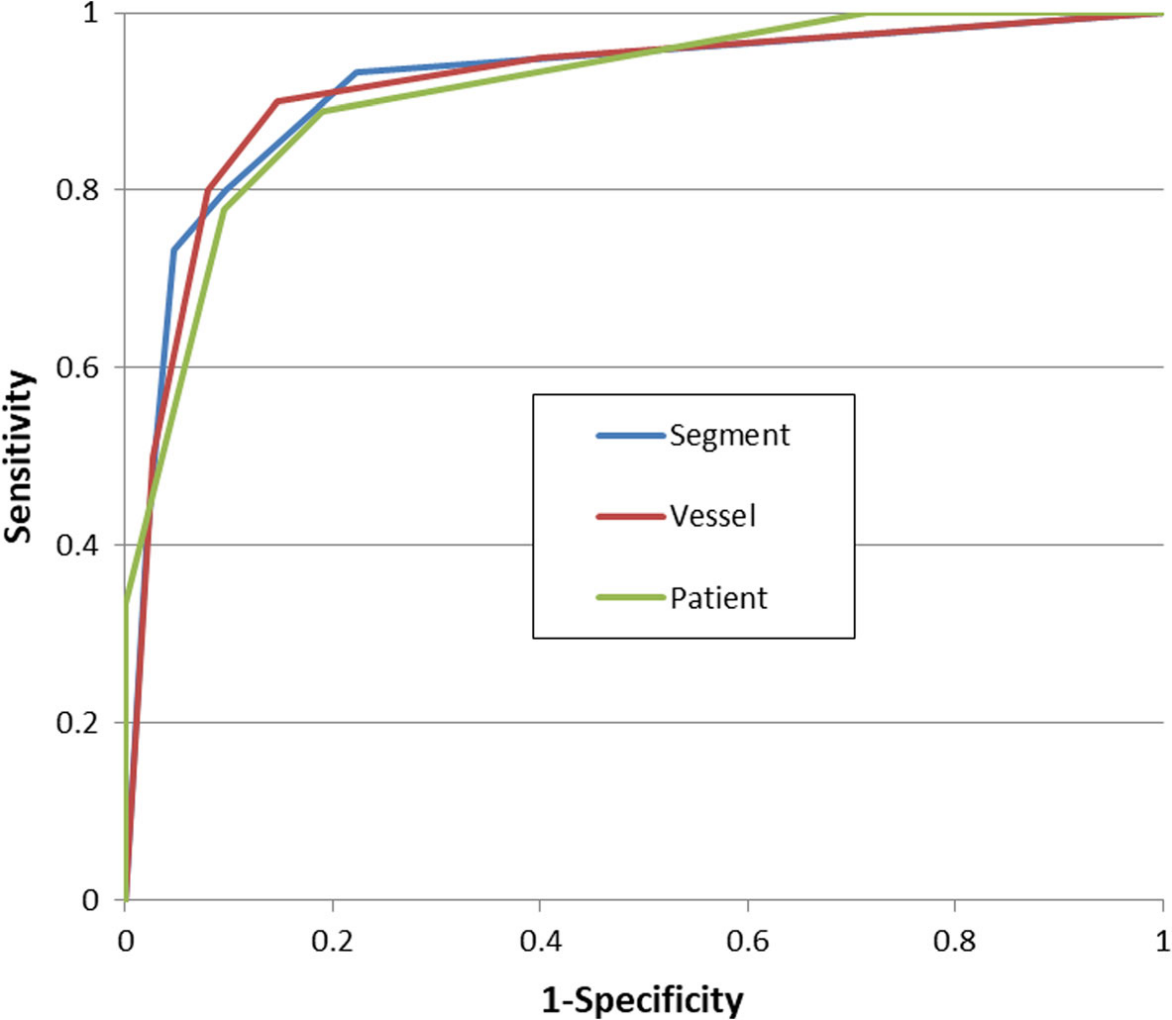
A receiver operator characteristic curves of iNAV CMRA for detecting significant coronary artery stenosis

**Fig. 5:**
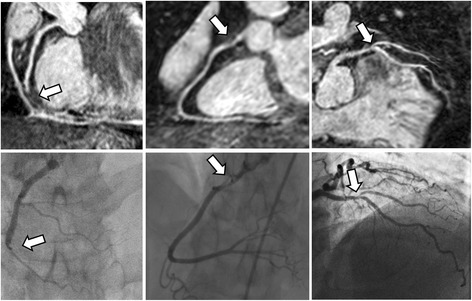
Images from three patients with coronary artery disease, diagnosed using coronary magnetic resonance angiography (top row) and confirmed with coronary x-ray angiography (bottom row)

Seven patients (24%) were found to have significant CAD based on coronary x-ray angiography. This included 8 diseased proximal segments, four diseased middle segments and three diseased distal segments. The receiver-operator characteristics curves for the CMRA per patient, vessel and segment are shown in Fig. 4. The per-patient, vessel and segment area under the curve was 0.91% (95% CI: 79% to 100%), 93% (95% CI: 81% to 100%) and 92% (95% CI: 84% to 99%), respectively. CMRA was able to detect significant CAD in 6 out of 7 patients (86%), 8 out of 10 vessels (80%), and 11 out of 15 segments (73%). The sensitivity, specificity, positive predictive value and negative predictive value for the-per patient, vessel and segmental analysis are summarized in Table 2. Example images from three patients with coronary artery disease, where the diagnosis was identified from the CMRA and confirmed in the coronary X-ray angiography, are shown in Fig. 5.

**Table 2:** Diagnostic performance. Data are % (raw data) [95% confidence interval]. PPV = positive predictive value; NPV = negative predictive values

	Patient	Vessel	Segment
Sensitivity	86 (6/7) [42–99]	80 (8/10) [44–97]	73 (11/15) [45–92]
Specificity	83 (21/24) [62–95]	92 (68/72) [83–97]	95 (227/238) [92–98]
PPV	60 (6/10) [37–79]	57 (8/14) [34–75]	50 (11/22) [34–66]
NPV	95 (20/21) [76–99]	97 (68/70) [91 99]	98 (227/231) [96–99]

## Discussion

In this work, we have evaluated a new approach for respiratory motion compensated CMRA using image navigator motion correction and gating. Compared to conventional CMRA motion compensation using a diaphragmatic navigator, the proposed approach reduces operator dependence as no dedicated respiratory navigator scan planning is required. The inline motion compensation allowed CMRA reconstruction at the scanner console and visualization of coronary arteries to aid diagnosis without interrupting the clinical workflow. A high percentage (241 of 253; 95%) of coronary artery segments was of diagnostic image quality, suggesting the proposed iNAV CMRA approach is a robust and reliable tool with additive clinical value.

In recent years, there have been a few studies in patients with CAD using CMRA with conventional respiratory motion compensation. In a multi-center trial Kato et al. [19] reported a per-patient sensitivity and specificity of 88% and 72%, respectively, using 1.5 T scanners with Cartesian bSSFP acquisition and a spatial resolution similar to iNAV CMRA. Yang et al. [20] obtained a sensitivity of 94% and specificity of 82% using a 3 T scanner and slow-infusion contrast enhanced gradient echo acquisition. While the diagnostic performance in these studies was comparable to iNAV CMRA, the scan time was approximately 2 min longer, with a large distribution of values reflecting the unpredictability of scan times using the conventional navigator approach. Furthermore, the reported failure rate in these studies was 8–10%, while the completion rate of iNAV CMRA was 100%.

A further two studies have reported on the use of advanced respiratory motion compensation strategies in patients with CAD. Piccini et al. used a radial k-space trajectory with translational correction and 100% gating efficiency, and obtained a per-patient sensitivity and specificity of 71% and 62%, respectively [21]. Differences in navigator acquisition approach may explain the differences in diagnostic performance compared to iNAV CMRA, where Piccini et al. used a one-dimensional projection navigator to detect motion, which may include static tissue leading to motion under-estimation. In comparison, the spatially resolved iNAV avoids this problem, allowing for accurate motion estimation even with only a few phase encoding steps [16]. Furthermore, a constant 50% gating efficiency was implemented for iNAV CMRA. This effectively discards the most motion corrupted data which may have been corrupted by large translations and n’on-rigid motion. Despite the scan time penalty introduced by the respiratory gating, the mean scan time using iNAV (7:21 min:sec) was shorter compared to Piccini et al. (7:50 min:sec) This is likely due to the use of parallel imaging with a factor of 2.5, as well as the lower spatial resolution. Similar to Piccini et al., He et al. [18] used a radial trajectory with 100% respiratory gating efficiency but more advanced 3D affine motion correction and reported a per-patient sensitivity and specificity was 96% and 69%, respectively. Apart from the technical differences in strategies for respiratory motion correction, a higher field strength (3 T) and higher spatial resolution (1 mm isotropic), He et al. used a stricter exclusion criteria which encompassed patients with arrhythmia. However, this criterion was not applied in the current study and affected the diagnostic performance as a majority of non-diagnostic segments were found in two patients with significant arrhythmia, which led to false positive diagnosis, lower specificity and PPV. Compared to the technique developed by He et al. iNAV CMRA does not require offline, retrospective post-processing. To account for non-rigid motion without the use of respiratory gating could involve implementing image registration and correction with more degrees of freedom, such as affine or non-linear motion models. However, this is technically challenging to perform in real-time due to the increased computational complexity. Furthermore, the spatial resolution of the navigator also has to be sufficiently high to capture this motion, whereas the proposed iNAV has limited resolution in LR direction and is a projection of the heart in anterior-posterior direction. Recently, techniques have been proposed to enable motion estimation from high resolution navigators, by combining data from multiple cardiac cycles but the same respiratory state [18, 22–24]. A drawback of attempting to estimate and correct motion with more degrees of freedom is the inclusion of additional noise associated with these measurements. Robust registration algorithms are required to minimize this source of noise. The use of global motion models to correct for all respiratory motion also risk introducing motion artifacts arising from tissue within the FOV which has different respiratory motion characteristics than the heart.

A robust CMRA sequence with short and predictable scan time would allow integrating CMRA into routine CMR scanning. CMRA has been used as part of a standard protocol in scientific studies [25] but not implemented in clinical scanning due to unpredictable scan time and image quality. Integrating CMRA would have a number of advantages in comparison to invasive angiography or coronary CT angiography, as it is non-invasive, not limited by the presence of coronary calcifications, does not use ionizing radiation and uses well tolerated and not nephrotoxic contrast agents. Additionally, the visualization of coronary morphology using CMRA can be integrated into a comprehensive evaluation of patients with angina symptoms, which also integrates optimal assessment of cardiac function, myocardial perfusion and viability and tissue characterization [26], therefore extending the diagnostic spectrum to include other causes of angina-like symptomatology [27]. Since the presence of stents systematically leads to metal artifacts and prohibiting the assessment of the coronary lumen these patients may be less well suited. The high negative predictive value of CMRA (similar to CTA studies) may increase the utilization of CMR as a first line tool in patients with low to intermediate pretest likelihood for significant coronary artery disease. Due to the excellent performance of CMR perfusion imaging in diagnosing significant ischemia [25] as well as guide patient management [28] CMR would offer a complete package in a wide range of patients.

This study has a number of limitations. It contains relatively few patients with low prevalence of CAD (24%) and thus resulted in a wide 95% confidence interval. From a technical perspective the technique is currently incompatible with the conventional arrhythmia rejection algorithm which renders it unsuitable for patients with frequent arrhythmias.

## Conclusions

In this work we have demonstrated that iNAV is a robust approach for mitigating motion artefacts for CMRA in patients with suspected CAD. Due to the short and predictable scan time it can add clinical value as a part of a comprehensive CAD assessment protocol.

## Abbreviations

2D: Two-dimensional
3D: Three-dimensional
bSSFP: Balanced steady-state free precession
CAD: Coronary artery disease
CI: Confidence interval
CMRA: Coronary magnetic resonance angiography
CRUISE: Constant respiratory efficiency using single end-expiratory threshold
FOV: Field-of-view
iNAV: With image-based navigation
LAD: Left anterior descending coronary artery
LCX: Left circumflex coronary artery
LM: Left main coronary artery
RCA: Right coronary artery
